# A riddle wrapped in an enigma: acute kidney injury in a girl with Crohn’s disease: Questions

**DOI:** 10.1007/s00467-020-04537-z

**Published:** 2020-03-30

**Authors:** Lilach C. Regev, Antonia H.M. Bouts, Jaap W. Groothoff, Joanna A.E. van Wijk, Michiel van Wijk, Paul van der Valk, Arend Bökenkamp

**Affiliations:** 1 Edmond and Lily Safra Children’s Hospital, Sheba Medical Center, Ramat-Gan, Israel; 2 Department of Pediatric Nephrology, Emma Children’s Hospital, Amsterdam University Medical Centers, Amsterdam, The Netherlands; 3 Department of Pediatric Gastroenterology and Nutrition, Emma Children’s Hospital, Amsterdam University Medical Centers, Amsterdam, The Netherlands; 4 Department of Pathology, location VU medical center, Amsterdam University Medical Centers, Amsterdam, The Netherlands

## Case presentation

A 15-year-old girl with a recent diagnosis of Crohn’s disease was admitted to Emma Children’s Hospital with complaints of right lower quadrant abdominal pain, nausea, anorexia, and reduced urine output with anuria for the last 8 h. She had no fever and no diarrhea. She had lost 5 kg in weight during the past month but put on again 1.5 kg in the last week.

On admission, her blood pressure was mildly elevated to 127/90 mmHg (95–99th percentile), with a heart rate of 90 BPM. Her height was 155 cm and weight 41.5 kg. On physical examination, she was well appearing, with right lower quadrant and bilateral flank tenderness. She had no rash, no edema, and normal capillary refill. Laboratory tests showed a hemoglobin of 10.5 mg/dL (5.2 mmol/l), thrombocytosis of 930,000 per microliter, leukocytosis of 12,300 per microliter, with an absolute neutrophil count of 9800 per microliter, and normal eosinophil count (40 per microliter). C-reactive protein was 38 mg/L. Serum creatinine was elevated to 2.5 mg/dL (220 micromol/L), blood urea nitrogen was normal (10.3 mg/L, i.e., 3.7 mmol/L). Albumin was 3.8 g/dL (38 g/L), phosphorus 5.1 mg/dL (1.65 mmol/L), sodium 135 mmol/L, and potassium 4.1 mmol/L. Blood gases were normal with a bicarbonate of 24 mmol/L.

Her urine sediment was positive for leukocytes (244 per microliter) and epithelial cells, with no RBC, casts, or crystals. There was moderate proteinuria with a protein/creatinine ratio of 0.9 mg/mg (104 mg/mmol). Urine sodium was < 20 mmol/L, and urine urea 43 mmol/L, with fractional excretions of 0.6% and 43%, respectively. Urine osmolality was 118 mOsmol/kg. Abdominal ultrasound showed stable ileocecal inflammation and normal renal size and structure. Urine culture was sterile.

## Past medical history

The girl had been diagnosed with steroid-resistant Crohn’s disease 2 months before admission. At that time, kidney function had been normal (creatinine 0.65 mg/dL, i.e., 57 μmol/L). Two weeks before admission, she had presented at the emergency department with fever, and very severe right lower quadrant abdominal pain. Ultrasound examination suggested abscess formation, for which she was started on IV antibiotics. On the next day, an abdominal MRI with enterography had not confirmed this but did show signs of ileocecal inflammation. Therefore, IV antibiotics were discontinued and she was started on oral antibiotic therapy (ciprofloxacin and metronidazole) as an add-on to the treatment for Crohn’s disease.

During the month prior to admission, she had received the following medications: (i) Analgesics: ibuprofen for 3 days 4 weeks before admission, tramadol and paracetamol daily until admission. (ii) Anti-inflammatory and immunosuppressive medication: infliximab on days 23 and 9 before admission, azathioprine for the last 6 days before admission. (iii) Antibiotics: gentamicin and amoxicillin/clavulanic acid on days 16 and 15 before admission, metronidazole and ciprofloxacin for the last 14 days until admission. She had also taken 3 drops of home-made cannabinoid oil on days 3 and 2 before admission.

## Questions:

1. Does this patient have AKI?
2. How do you explain the discrepancy between the high creatinine and normal BUN?
3. What is the differential diagnosis of her renal condition?
4. What is the most likely type of renal injury in this case?

